# Long-term storage does not impact the quality of cryopreserved human ovarian tissue

**DOI:** 10.1186/s13048-016-0261-8

**Published:** 2016-08-24

**Authors:** Raffaella Fabbri, Maria Macciocca, Rossella Vicenti, Gianandrea Pasquinelli, Giacomo Caprara, Sabrina Valente, Renato Seracchioli, Roberto Paradisi

**Affiliations:** 1 Gynecology and Physiopathology of Human Reproductive Unit, Department of Medical and Surgical Sciences, University of Bologna, S. Orsola-Malpighi Hospital of Bologna, via Massarenti 13, 40138 Bologna, Italy; 2 Surgical Pathology, Department of Experimental, Diagnostic and Speciality Medicine, University of Bologna, S. Orsola-Malpighi Hospital, Bologna, Italy; 3 Histopathological and Molecular Diagnostic Unit of Solid Organ and Transplant, S. Orsola-Malpighi Hospital, Bologna, Italy

**Keywords:** Human ovarian tissue cryopreservation, Long-term storage, Tissue quality

## Abstract

**Background:**

Ovarian tissue cryopreservation is an emerging technique, also addressed to very young cancer patients, for whom it is not possible to perform an ovarian stimulation for oocytes freezing, before gonadotoxic treatment. In this cases, ovarian tissue must be cryopreserved for a long period of time and it is very important to know if it maintains fertility function after a long period of storage. Here we aimed to assess the effect of long-term storage on preservation and viability of cryopreserved human ovarian tissue.

**Methods:**

Descriptive study of three cases of cancer patients whose cryopreserved ovarian tissue remained stored for 18 years. Long-term stored tissue was examined by histological and immunohistochemical analysis, transmission electron microscopy, TUNEL assay and LIVE/DEAD viability/citotoxicity test.

**Results:**

Ovarian tissue stored for 18 years showed a good morphology. Follicles presented negative staining for estrogen and progesterone receptors, positive staining for ki67 in granulosa cells and/or oocytes and for bcl2 in granulosa cells. Regarding stroma, patch/focal positive expression was found for estrogen receptor and ki67, diffusely positive expression for progesterone receptor and bcl2. After long-term storage, ultrastructural examination showed sub-cellular integrity of follicles and interstitial oedema foci. No apoptosis was observable by TUNEL assay. Stromal cell viability remained >97 % during the culture period.

**Conclusion:**

The evaluation of different aspects of the tissue provides evidence that the storage time does not impact on tissue quality and gives hope especially to cancer girls, whose tissues could remain cryopreserved for a very long time.

## Background

Recent advances in the diagnosis of cancer and the availability of new protocols of chemo- and radio-therapy have significantly increased the life span of children, adolescents and adults with cancer [1]. Unfortunately, these treatments are gonadotoxic and can severely affect or totally destroy the reproductive potential of patients [2].

Ovarian tissue cryopreservation represents a promising strategy to preserve reproductive function and steroidogenic activity in patients with a high risk of premature ovarian failure. This technique can be performed at any time in the ovarian cycle, thereby avoiding delays in starting therapy; it is particularly indicated in patients with hormone sensitive tumors and it is the only available option to preserve ovarian function in prepubertal girls [3, 4].

At remission of disease, the cryopreserved ovarian tissue can be reimplanted in the patients to restore the ovarian function. Many patients remained interested in maintaining cryostorage of their ovarian tissue beyond an initial 5-year period [5] as ovarian tissue cryopreservation avoids the sequelae of preterm menopause and allows to postpone child bearing until early 40 years. The storage time of the ovarian tissue becomes even longer in youngest patients, for example prepubertal patients, at the time of the cryopreservation.

To date, studies on ovarian tissue storage are limited in numbers [6] and little is known about the influence of several years of storage on integrity and viability of the cryopreserved ovarian tissue.

The aim of this study was to evaluate the effect of 18 years of storage on preservation and viability of ovarian tissue cryopreserved by slow-freezing/rapid-thawing protocol.

## Methods

### Patients

The study was performed on the ovarian tissue of three patients: patient 1, 32 years suffering from right ovarian adenocarcinoma (in this patient an ovarian biopsy was retrieved from the healthy left ovary); patient 2, 36 years suffering from breast cancer; patient 3, 31 years suffering from colon cancer. The patients cryopreserved the ovarian tissue at Gynecology and Physiopathology of Human Reproductive Unit of S. Orsola-Malpighi Hospital of Bologna (Italy) in 1997.

### Study design

The ovarian tissue of the three patients was harvested by laparoscopy and treated as reported in Fabbri et al. [7]: a) one or two cortical strips per patient were immediately fixed in formalin for histological and immunohistochemical analysis (control fresh tissue) and the remaining cortical strips were cryopreserved by slow-freezing protocol; b) after short-term storage (120 days), one or two cortical strips per patient were thawed and fixed for histological and immunohistochemical analysis, to verify the efficiency of the ovarian tissue cryopreservation procedure.

In the present study, one or two cortical strips of the same three patients were thawed after long-term storage (18 years) and evaluated for:

- Ovarian tissue quality by histological and ultrastructural analysis to assess the morphological features of follicles and stromal cells, similarly to what performed in Fabbri et al. [7];
- Maintenance of ovarian potential ability to respond to reproductive hormones by estrogen and progesteron receptor antibodies in immunohistochemistry, since the steroids are well-recognized regulators of folliculogenesis;
- Preservation of cell proliferation and anti-apoptotic activity as determined by anti-ki67 and anti-Bcl2 antibodies in immunohistochemistry;
- Incidence of apoptosis phenomena by TUNEL assay that highlights DNA fragmentation results from apoptotic signaling cascades;
- In-vitro cell viability by LIVE/DEAD viability/citotoxicity test that discriminates live from dead cells by simultaneously staining with green-fluorescent calcein-AM, to indicate intracellular esterase activity, and with red-fluorescent ethidium homodimer-1, to indicate loss of plasma membrane integrity.

### Slow-freezing/rapid-thawing protocol

Ovarian tissue was cryopreserved using a slow-freezing protocol described by Fabbri et al. [7]. In brief, ovarian cortical strips were placed in plastic cryovials (Intermed Nunc Cryotubes, Roskilde, Denmark) containing 1.8 ml of “freezing solution” consisting of 1.5 M 1,2-propanediol (PROH - Fluka Chemica, Sigma Aldrich SrL; Milan, Italy), 0.2 M sucrose (Fluka Chemica, Sigma Aldrich SrL; Milan, Italy), and 30 % heat-inactivated human serum (HS - provided by the Transfusion Centre of S. Orsola-Malpighi Hospital) in Dulbecco’s phosphate-buffered saline (DPBS-Gibco, Life Technologies, Paisley, Scotland) and maintained at 0 °C in an ice bath. The cryovials were transferred to a rolling system (Continents instrument, Amityville, USA) for 30 min at 4 °C to allow the cryoprotectant to enter the tissue; they were then cooled in a programmable freezer (Planer Kryo 10/1,7 Series III, SAPIO Life, Milan, Italy) allowing the gradual reduction of the temperature from 0 to −140 °C. The starting temperature was 0 °C; then it was slowly reduced to −9 °C at a rate of 2 °C/min. Ice nucleation was induced manually at −9 °C (seeding). After a holding time of 10 min at −9 °C, the cryovials were cooled slowly to −40 °C at a rate of 0.3 °C/min and then rapidly to −140 °C at a rate of 10 °C/min. After 10 min of temperature stabilization, the cryovials were transferred into liquid nitrogen tanks and stored until thawing.

Frozen ovarian tissue was thawed using a modified rapid-thawing protocol described by Fabbri et al. [8]. In brief, the cryovials were air-warmed for 30 s and then immersed into 37 °C water bath for 2 min. The cryoprotectants were removed at 4 °C by four-stepwise dilution: (i) 0.76 M PROH, 0.175 M sucrose, 30 % HS in DPBS for 5 min; (ii) 0.26 M PROH, 0.175 M sucrose, 30 % HS in DPBS for an additional 5 min; (iii) 0.175 M sucrose, 30 % HS in DPBS for 10 min and finally, (iv) DPBS supplemented with 30 % HS for 20 min.

### Histological - immunohistochemical - TUNEL analysis

Frozen/thawed cortical strips were embedded in paraffin blocks and nine 4 μm thickness serial sections were obtained per block: the first, third and seventh were stained with hematoxylin and eosin (Merck, Darmstadt, Germany), to assess the morphological features of follicles and stromal cells. Follicle development stage was establish according to Gougeon classification [9].

The second, fourth and sixth sections were used for immunohistochemistry, as described in Fabbri et al. [7], and incubated overnight at 4 °C with the following primary antibodies: anti-estrogen receptor (ER) clone 1D5 (1:20, Bio Genex, San Ramon, CA), anti-progesterone receptor (PR) clone 1A6 (1:20, Bio Genex, San Ramon, CA), anti-proliferating antigen Ki67 (1:80, Bio Genex, San Ramon, CA), anti-protein Bcl2 (1:80, Dako, Carpinteria, CA). An En Vision monoclonal immunoenzymatic system was used for immunnohistochemical detection (Dako). The reaction was developed in 3,3-diaminobenzidine (DAB, Sigma, St. Louis, MO, USA). Finally, the sections were counterstained with Mayer’s hematoxylin for 10 s, dehydrated, and mounted in Eukitt. Control procedures were undertaken simultaneously to ensure the specificity of immunostaining [7]. Sections without primary antibodies were used as a negative control and sections of human breast cancer were used as a positive control. Follicle and stromal positivity for the primary antibodies were evaluated at 200X magnification under a Leitz microscope. Follicles with oocyte nucleus and at least one stained granulosa cell were considered positive.

The fifth, eighth and ninth sections were collected (Superfrost-plus slides Menzel-Gläser, Braunschweig, Germany) for terminal deoxynucleotidyl transferase dUTP nick end labeling (TUNEL) analysis using the cell death detection kit conjungated with horseradish peroxidase (Roche, Mannheim, Germany), according to the manufacturer’s instructions. Briefly, after deparaffination, rehydratation and washing with phosphate-buffered saline, four to six sections were immersed in 10 mM citrate buffer and underwent antigen retrieval in a microwave for 5 min (350 W). Then the slides were incubated with 50 mL of TUNEL reaction mixture and incubated at 37 °C for 60 min in the dark inside a humidified chamber.

As positive control, sections were incubated with DNase I (3000U/ml in 50 mM Tris–HCl, pH 7.5, 1 mg/ml BSA) for 10 min at 15–25 °C to induce DNA strand breaks, prior to labeling procedure; as negative control, sections were incubated with label solution only (without terminal transferase) instead of TUNEL reaction mixture.

The apoptotic signal was recorded as positive when dUTP stained the nucleus brown. Follicles were considered damaged when the oocyte nucleus and/or more than two of granulosa cells were stained in brown by dUTP. The number of apoptotic stromal cells was analyzed in a 100 mm^2^ (randomly assigned in the sections) and was counted three times and then averaged. The percentage of apoptotic cells was calculated as follows: apoptotic cell number/total cell number × 100 and quantified in a double blind fashion using a Leitz Diaplan light microscope equipped with a CCD JVC video camera. Digitized images were analyzed with Image ProPlus software.

All sections were examined on a blind fashion by two different pathologists.

### Ultrastructural analysis

Frozen/thawed (after long-term storage) cortical strips were fixed in a 4 % buffered paraformaldehyde solution at pH 7.4 overnight at 4 °C. After osmium tetroxide postfixation and alcohol dehydration, the samples were embedded in epoxy resin (Araldite, Fluka, Switzerland) and then cut using an ultramicrotome (Ultracut, Reichert, Vienna, Austria). Sixty-nm thick sections were collected on 200 mesh grids, stained with uranyl acetate followed by lead citrate and viewed using a Philips 410 T transmission electron microscope at 80 kV, in order to evaluate the integrity of oocytes, granulosa and stromal cells according to previously reported subcellular criteria [8].

### Viability/Cytotoxicity LIVE/DEAD assay

Frozen/thawed (after long-term storage) cortical strips were digested in an enzymatic solution containing Epicult-B Basal Medium (StemCell Technologies) and Collagenase/Hyaluronidase (3000 U/mL Collagenase, 1000 U/mL Hyaluronidase. Stemcell Technologies Milan, Italy) at 37 °C for 4 h. The digested cell solution was centrifuged at 200 g for 3 min and the pellet was re-suspended in 1 ml culture medium composed of α-MEM (Alpha Minimum Essential Medium, Sigma, Italy), 1 % ITS (5 μg/mL insulin, 5 μg/mL transferrin, 5 ng/mL selenite, Sigma, Italy), 20 % HS, 25 mmol/l NAC (N-Acetyl-cystein, Sigma, Italy), and antibiotics. Cells were then cultured in 2 ml of medium in sterile 35 mm petri dishes at 37 °C and 6 % CO2 for 15 days. Culture medium was replaced with pre-equilibrated fresh medium every 48 h.

20.000 cells were seeded on a sterile glass coverslip inside 35 mm petri dishes and cell viability was checked after 2 and 7 days of culture. At the end of culture period the cells were washed with Dulbecco’s phosphate-buffered saline (D-PBS) and treated with Viability/Cytotoxicity LIVE/DEAD Kit (Molecular Probes, Invitrogen, Milan, Italy) based on the simultaneous determination of live and dead cells with two probes, calcein-AM and ethidium homodimer-1(EthD-1). 0.5 mM Calcein-AM and 4 mM EthD-1 were added to culture dishes for 30 min at room temperature. Following incubation, the coverlisp was cleaned with D-PBS, mounted on the microscope slide and immediately viewed under the fluorescent microscope (Leica CTR6000, Leica Microsystems, Germany).

## Results

### Histological - immunohistochemical - TUNEL analysis

**Fig 1:**
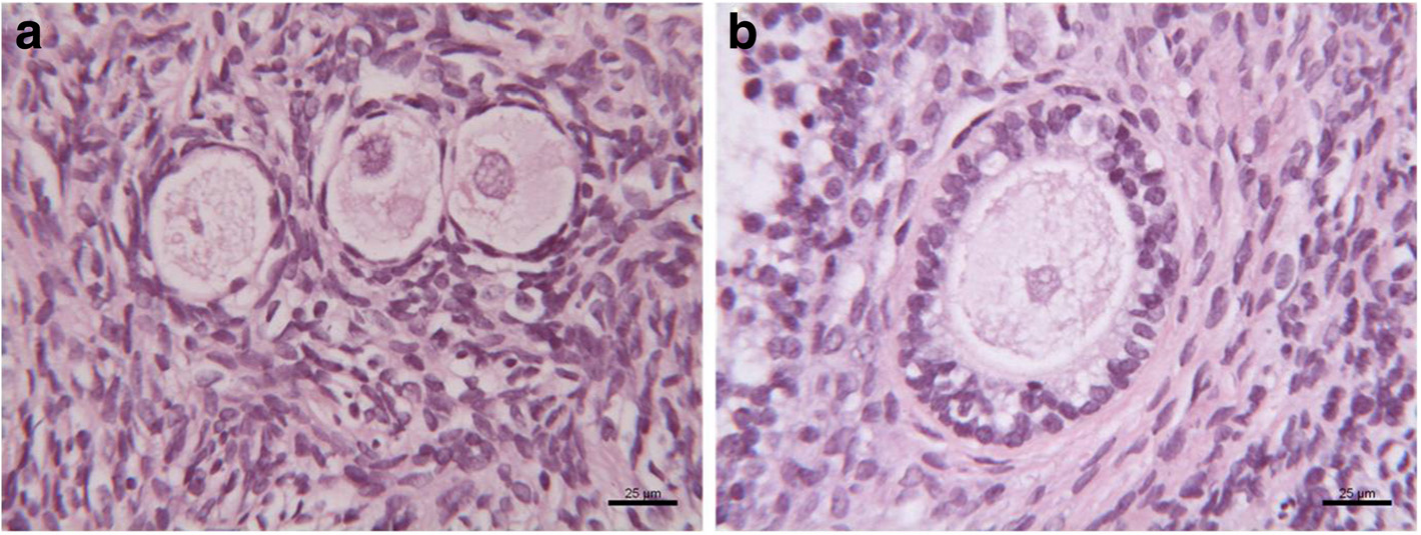
Morphological appearance of human cryopreserved ovarian tissue after long-term storage. **a** Primordial follicles, **b** secondary follicle

After long-term storage, frozen/thawed ovarian tissue appeared well-preserved. Most of follicles were primordial (Fig. 1a); follicles at advanced stage of development (primary, secondary, pre-antral) were also present (Fig. 1b). Stromal cells showed an intact architecture: oval- to spindle-shaped nucleus with dispersed chromatin; no interstitial oedema or vacuoles were present (Fig. 1a,b).

**Fig. 2:**
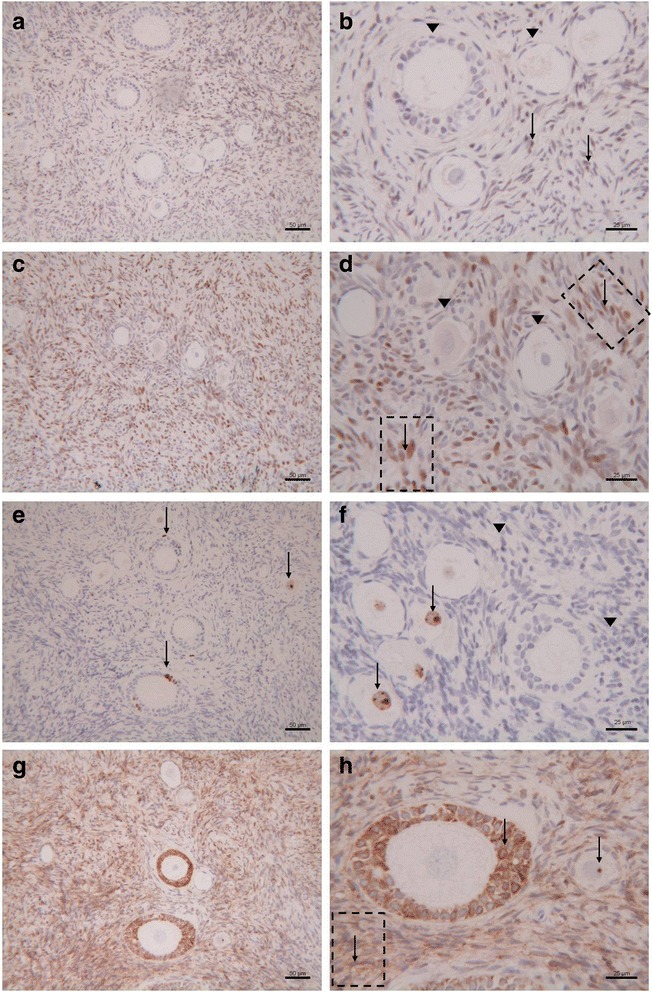
Immunohistochemical staining for ER (**a**, **b**), PR (**c**, **d**), ki-67 (**e**, **f**) and bcl2 (**g**, **h**) in follicles and stroma of human cryopreserved ovarian tissue after long-term storage. () Negative staining; () Positive staining

Results of immunohistochemical analysis showed a similar staining pattern distribution in the three patients. In particular, estrogen receptors (ER) were negative in all examined follicles and showed a patch/focal expression (<10 % positive cells) in stromal cells (Fig. 2a,b). Akin progesterone receptors (PR) were negative in follicles but diffusely positive (>50 % positive cells) in stromal cells (Fig. 2c,d). Concerning ki67 protein, a positive nuclear staining was found in both the granulosa cells and/or oocytes in 44 % (21/48) of the frozen/thawed follicles, while no positive staining was observed in stromal tissue (Fig. 2e,f). As to bcl2 protein (bcl2), positive staining was found in the granulosa cells of secondary/preantral follicles, but not in the oocytes. Stromal tissue was diffusely bcl2 stained (Fig. 2g,h).

Analogous results were observed in fresh and frozen/thawed (after short term storage) tissue of the same three patients and reported in Fabbri et al. [7].

**Fig. 3:**
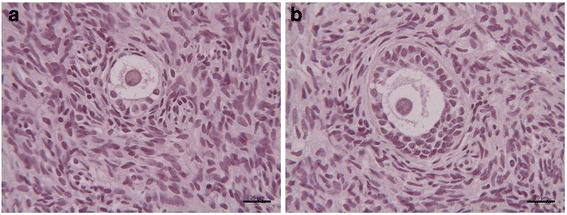
TUNEL staining of follicles and stroma of human cryopreserved ovarian tissue after long-term storage. **a** Primordial follicle; **b** Secondary follicle. No apoptosis incidence in primordial (**a**) and secondary follicles (**b**) as well as in the stroma (**a**, **b**)

No TUNEL-positive cells were found in follicles at primordial stage (Fig. 3a) and at more advanced stage of development (Fig. 3b) as well as in the stroma (Fig. 3a,b) of frozen/thawed tissue after long-term storage.

### Ultrastructural analysis

**Fig. 4:**
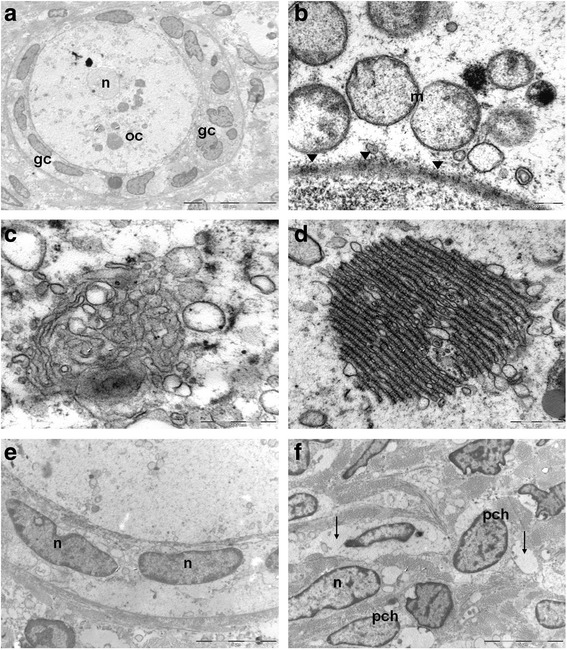
Transmission electron microscopy of human cryopreserved ovarian tissue after long-term storage. **A** primordial/primary follicle: the oocyte shows a nucleus (n) having finely dispersed chromatin and cytoplasm (oc) rich in organelles; a layer of flattened/cuboidal granulosa cells (gc) surrounding oocyte cytoplasm; **B** perinuclear aggregates of rounded mitochondria (m) having moderate electron dense matrices, () nuclear pores; **C** Golgi apparatus; **D** annulate lamellae consist of membrane-bound cisternae traversed by pore complexes; **E** granulosa cells with oval shaped nuclei (n); **f** stromal cells with oval shaped nuclei (n) and slight band of peripherically condensed chromatin (pch). Empty interstitial areas are detectable ()

TEM examination of frozen/thawed follicles after long-term storage, showed large oocytes with regular nuclei and finely dispersed chromatin (Fig. 4a), intact nuclear membranes and nuclear pores (Fig. 4b). Mitochondria, free ribosomes, rough and smooth endoplasmic reticulum, Golgi apparatus and occasional lipid inclusion and lipofuscin bodies were observed in the oocyte cytoplasm. In particular, mitochondria appeared typically round, with a low-density matrix, clustered around a dense and amorphous intermitochondrial substance (Fig. 4b); the Golgi apparatus was well developed, consisting of numerous flattened cisternae and vesicles (Fig. 4c); stacks of annulate lamellae were recorded in the nucleus proximity (Fig. 4d). Granulosa cells with flat nuclei with dispersed euchromatin were adherent to the oocyte (Fig. 4e).

Stroma cells showing large euchromatic oval nuclei and nuclear clefts (Fig. 4f) were embedded in a collagenous matrix. Empty clear stromal areas were seen and considered a consequence of cryodamage.

### Viability/Cytotoxicity LIVE/DEAD Kit assay

**Fig. 5:**
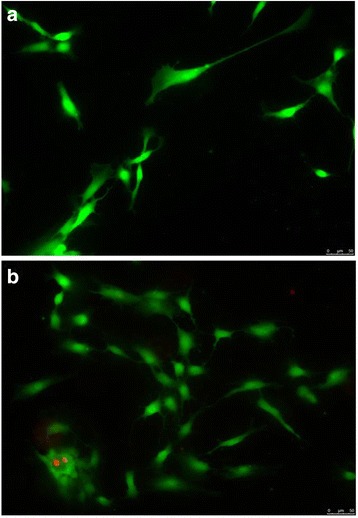
LIVE/DEAD fluorescence staining of stromal cells isolated from human cryopreserved ovarian tissue after long-term storage and cultured for 2 days (**a**) and 7 days (**b**). Green cells are alive cells, red cells are dead cells

The Viability/Cytotoxicity LIVE/DEAD assay performed on stromal cells enzymatically isolated from frozen/thawed tissue after long-term storage showed viable cells after 2 (Fig. 5a) and 7 (Fig. 5b) days of culture; these cells had the ability to grow without the growth factor supplementation. During the culture period the viability of cells remained always >97 %.

## Discussion

The ovarian tissue cryopreservation is a complex technique that requires experience and validation [8, 10, 11]. Although reproductive performance and recovery of regular cyclic function after transplantation of cryopreserved ovarian tissue are encouraging [12–14], several studies report a decreased reproductive potential of frozen ovarian tissue transplantation than fresh one [15–18]. It might be likely related to the cryopreservation procedure that determine oocyte/stroma damage [19, 20] or to ischemic injury to follicles, caused by the freeze-thaw procedures in cryopreserved ovarian grafts [21–23].

A recent study investigated the influence of the storage time (30 and 180 days) on the magnitude of tissue damage [6]. The authors found that the short-term storage of ovarian tissue did not appear to compromise follicle integrity and steroidogenic capacity of the tissue.

However, it must be considered that usually the ovarian tissue remains frozen for a very longer time period. In fact, the request to re-implant the cryopreserved ovarian tissue usually occurs after at least five years from the end of therapies, when oncologists declare the patient completely healed [5]. This period may dragging on further in the case of diseases that require prolonged treatments, or in the case of pediatric patients.

As to date there are no reports describing the impact of long-term storage on cryopreserved ovarian tissue, the present study aimed to investigate the morpho-functional features of frozen ovarian tissue stored for 18 years.

The efficiency of cryopreservation procedure was previously determined by the comparison of fresh and frozen/thawed tissue after short-term storage as reported in Fabbri et al. [7]. The success of freezing/thawing protocol was evidenced by a good stromal and follicular morphology in the specimens, maintenance of ovarian antigenicity (ER and PR expression), of cellular proliferation (ki67 expression), and of anti-apoptotic index (Bcl-2 expression). During the last 12 years our research group worked in order to optimize the cryopreservation protocol to improve the preservation of ovarian tissue by checking the seeding temperature, which is a crucial step of slow-freezing/rapid-thawing protocol, and by testing different concentrations of cryoprotectants and protein support. The best preservation of morphological characteristics was obtained using the freezing-thawing protocol in which the seeding was performed at −9 °C [24, 25] and the concentrations of cryoprotectants were 1.26 M PROH and 0.175 M sucrose added with 30 % human serum [8]. This cryopreservation protocol was further modified performing all thawing steps at 4 °C rather than at room temperature. The ultrastructural analysis showed a reduction of interstitial oedema and vacuolation in the stroma when the cryoprotectants were removed at lowest temperature (data not published). This modified freezing-thawing protocol is now in use at our Centre.

In the present study we evaluated the effect of long-term storage on tissue preservation by using the same analysis performed in Fabbri et al. [7]. Histological analysis showed a well-preserved morphology of the single tissue components: oocytes, granulosa and stromal cells. In all follicles there was no specific positive staining for estrogen and progesterone receptors. According to our previous study [7], this result could be related to the follicle size composition seen in our samples: in fact the samples analyzed contained primordial, intermediary, primary and secondary, that do not express ER and PR [26]. ER expression is evidenced in antral and pre-ovulatory follicles, before and during the luteinizing hormone (LH) surge; PR expression is evidenced in pre-ovulatory follicles during the LH surge and in luteinized granulosa cells after ovulation. On the contrary, a positive staining for ER (focal) and for PR (diffuse) was observed in the stroma. Expression of steroid receptors in the stroma tissue around small follicles has also been consistently observed in other species [27–30]. Steroids might indirectly regulate the small follicles by modulating the stromal cell-follicular interactions as well as through direct regulation of follicular cells. One potential mechanism by which steroids may indirectly regulate early follicular growth through interaction with the stromal cells is through regulation of small growing follicle vascularization [31, 32].

We also assessed the proliferative status of the ovarian cell population by monoclonal ki67 immunostaining. The monoclonal ki67 antibody recognizes a nuclear antigen which is expressed in all stage of the cell cycle except G0, so it represents a good index of the capacity of cryopreserved tissue to proliferate after thawing. A positive staining was found in the most of follicles, including oocytes and granulosa cells, suggesting that follicles could resume the mitotic cycle and subsequently grow. No positive staining was found in stromal cells.

The high percentage of positivity for anti-apoptotic bcl2 protein in granulosa cells as well as in stromal cells also indicates the good preservation of tissue after 18 years of storage.

The comparison of current results with those of fresh and short-term stored tissue appeared very encouraging and suggested that storage time does not influence tissue preservation, antigenicity and proliferative potential.

In order to provide more detailed examination of ovarian tissue cryopreserved and stored for a long period, we also performed additional analysis, such as TUNEL assay, ultrastructural examination by transmission electron microscopy and in vitro-culture with LIVE/DEAD assay.

TUNEL assay is a standard technique for the detection of apoptosis in tissue sections. It is very sensitive and widely used, although it is prone to technical problems, mostly related to DNA strand-breaks associated with excessive levels of proteinase digestion, with fixation and processing procedures, or with the action of section cutting or other various pretreatments [33].

In the present study TUNEL assay, together with the immunostaining for the bcl2 anti-apoptosis-associated protein, allowed to have a countercheck of absence of apoptosis phenomena in the stored tissue.

Ultrastructural analysis allowed to assess the real integrity of sub-cellular components and to detect features of cryodamage not observable using light microscopy [34, 35]. In fact, transmission electron microscopy evidenced foci of interstitial oedema, likely due to tissue rehydratation occurring during the thawing procedure. This kind of cryodamage is often observed in the ovarian tissue cryopreserved by slow freezing/rapid thawing protocol [8, 35].

Since the morphological and antigenic integrity of the tissue immediately after thawing may not reflect its true state [34, 35], the viability of cryopreseved human ovarian tissue was demonstrated by follicle development, intense proliferation (by immunoexpression of cytokeratin and ki67) and neoangiogenesis (by immunoexpression of von Willebrand factor) in the frozen-thawed cultured tissue [24, 25, 36].

We evaluated the viability of frozen/thawed tissue using stromal cell culture by the LIVE/DEAD test. We demonstrated that stromal cells were viable and capable of growing in culture after the cryopreservation procedures and long-term storage.

Therefore, the evaluation of different aspects of the tissue gives a complete picture of the frozen/thawed tissue providing evidence that the time of storage does not impact on the morphological, antigenic preservation of the tissue including its proliferation potential and vitality.

In order to obtain a good tissue quality after cryopreservation it is very important that tissue is treated with extreme accuracy by experienced and highly qualified staff. Moreover any temperature change during tissue storage has to be prevented.

Of course only the recovery of ovarian function after replanting could give proof of the true capabilities of the cryopreserved and stored ovarian tissue. A recent report describes the efficiency of slow-freezing/rapid-thawing procedure to restore fertility after transplantation of ovarian tissue cryopreserved during childhood and storage for 10 years [13]. This paper shows for the first time the possibility of restoring endocrine function and obtaining the birth of a healthy baby from ovarian tissue harvested before puberty.

Unfortunately we could not have this confirmation since the patients have donated their tissue for research purpose. However, the follicular development in graft sites and the recovery of ovarian function for more than 2 years after transplantation of ovarian tissue cryopreserved using the slow-freezing/rapid-thawing protocol, described in Fabbri et al. [37], provides evidence of the feasibility of the procedure.

We also compared the slow-freezing/rapid-thawing protocol with the vitrification/warming protocol [38]. In agreement with Isachenko et al. [39], Rahimi et al. [40] and Oktem et al. [41], the slow-freezing/rapid-thawing gave better results than vitrification/warming. The ultrastructural analysis of vitrified follicles evidenced irregularly shaped oocyte nuclei with slightly thickened chromatin, broken or swollen mitochondria, vacuoles and clarification of cytoplasm. The laser scanning confocal microscopy analysis of bioenergetic/oxidative status indicated a severe functional damage in vitrified tissue, with reduced mitochondrial oxidative phosphorylation activity associated with a decrease in the mitochondrial capacity to synthesize reactive oxygen species.

Therefore, the conventional slow-freezing/rapid-thawing remains at this moment the goal standard technique for ovarian tissue preservation. Further studies will be absolutely necessary and essential to optimize the vitrification protocol in order to switch from the conventional freezing to vitrification for human ovarian tissue cryopreservation.

## Conclusions

The data of the present study support the validity of our cryopreservation and storage procedures and are very reassuring for ovarian tissue cryopreservation in prepubertal girls who will keep their ovarian tissue stored for long time. This finding is noteworthy for the safety of long-term banking in oncofertility programs.

Of course only the recovery of ovarian function after replanting could provide proof of the true capabilities of the cryopreserved and stored ovarian tissue.

## Abbreviations

ER: Estrogen receptor
PR: Progesterone receptor
TUNEL: Terminal deoxynucleotidyl transferase dUTP nick end labeling
α-MEM: Alpha minimum essential medium
ITS: Insulin, transferrin, selenite
HS: Human serum
NAC: N-acetyl-cystein
D-PBS: Dulbecco’s phosphate-buffered saline
LH: Luteinizing hormone
